# Integrated Field Evaluation of a Caseous Lymphadenitis Vaccine and Environmental Bacteriological Profiling in Commercial Goat Farms in South Korea

**DOI:** 10.3390/vaccines14080695

**Published:** 2026-08-12

**Authors:** Gyeong-Seo Park, Somin Lee, Minsung Park, Minseok Kim, Eunhui Lee, Sungmin Lee, Myung Hyee Kim, Byeong Yeal Jung, Chonghan Kim, Byoung Joo Seo

**Affiliations:** 1 Vaccine Lab, WOOGENE B&G Co., Ltd., Seoul 07299, Republic of Korea; gyeoungseopark@gmail.com (G.-S.P.); somin94@woogenebng.com (S.L.); pms0918@woogenebng.com (M.P.); mindol0406@gmail.com (M.K.); leh950709@woogenebng.com (E.L.); bacteria1004@naver.com (B.Y.J.); chonghan2004@naver.com (C.K.); 2 College of Veterinary Medicine, Jeonbuk National University, 79 Gobong-ro, Iksan 54596, Republic of Korea; 3 Soo Animal Hospital, Geochang 50141, Republic of Korea; mini7226@hanmail.net; 4 Korea Research Institute for Veterinary Biologics (KRIVB), Iksan 54531, Republic of Korea; kmh6841@hanmail.net

**Keywords:** caseous lymphadenitis, *Corynebacterium pseudotuberculosis*, goat vaccine, field trial, commercial goat farms, in-house ELISA, MALDI-TOF MS, bacteriological profiling, farm-level disease control

## Abstract

**Background/Objectives**: Caseous lymphadenitis (CLA), caused by *Corynebacterium pseudotuberculosis*, is difficult to control on goat farms because of subclinical infection, abscess rupture, environmental contamination, and repeated herd-level exposure. This study evaluated a Korean inactivated CLA vaccine under commercial goat farm conditions and combined vaccination outcomes with farm-level bacteriological profiling. **Methods**: Goats from three commercial farms were allocated according to baseline CLA serostatus and vaccination status into seropositive vaccinated, seronegative vaccinated, seropositive non-vaccinated, and seronegative non-vaccinated control groups. Vaccinated goats received two intramuscular doses at weeks 0 and 4. Clinical signs, external abscess occurrence, growth performance, CLA-specific antibody responses, and abscess bacterial loads were monitored. Farm-level microbial profiles were assessed using culture, MALDI-TOF MS, and targeted PCR. **Results**: Baseline antibody classification showed strong agreement between the commercial CLA ELISA and the in-house whole-bacterial IgG ELISA, with a Spearman’s rho of 0.89 (*p* < 0.001) and 96.7% classification agreement. Vaccination induced CLA-specific antibody responses in both assays and was not associated with a persistent reduction in growth performance. External abscesses were observed only in baseline seropositive goats. Abscesses occurred in 1/30 vaccinated and 2/15 non-vaccinated seropositive goats, but the difference was not statistically significant (*p* = 0.254). Microbial profiling showed farm-dependent detection patterns. **Conclusions**: The vaccine induced CLA-specific antibody responses (immunogenicity) with acceptable tolerability. The exploratory abscess endpoint was underpowered, and clinical protection was not confirmed; larger studies are needed.

## 1. Introduction

Caseous lymphadenitis (CLA) is difficult to control because it is not confined to clinically visible abscesses. In infected goat herds, *Corynebacterium pseudotuberculosis* persists through a cycle of subclinical infection, abscess rupture, environmental contamination, and repeated exposure of susceptible animals [1,2,3,4,5]. This herd-level persistence distinguishes CLA from acute bacterial infections that can be managed by treating affected individuals alone. Once the disease becomes established, clinical inspection, antimicrobial treatment, and sporadic removal of affected animals are rarely sufficient to interrupt transmission [1,2,3]. Therefore, the field value of a CLA vaccine should be assessed not only by serological responses under controlled conditions but also by its performance under commercial farm conditions.

CLA is a chronic infectious disease of sheep and goats caused by *C. pseudotuberculosis* biovar *ovis*, a facultative intracellular, Gram-positive bacterium associated with external and internal abscess formation [1,2,3,4]. The disease causes persistent productivity losses through reduced body condition, impaired carcass value, premature culling, and long-term herd contamination [1,2,3]. Its epidemiological importance is heightened by the high prevalence of subclinical infection and the limitations of early clinical detection [1,2]. Animals without obvious lesions may remain within the herd, while ruptured abscesses can release bacteria into housing environments, handling areas, bedding, soil, and animal-contact surfaces [5,6]. These features allow CLA to persist at the herd level even when the number of clinically affected animals appears limited.

The pathogenicity of *C. pseudotuberculosis* is linked to both host-adapted survival and environmental persistence. A lipid-rich cell wall supports resistance to host defenses, while phospholipase D contributes to vascular permeability, tissue invasion, and dissemination from the primary infection site to regional lymph nodes and internal organs [4,7,8]. Virulence-associated genes, including pld and iron-acquisition-related genes, have been widely detected in isolates from sheep and goats, supporting the conserved pathogenic potential of field strains [7,8,9]. In parallel, the organism can remain viable outside the host, especially when protected by purulent material, organic matter, or contaminated farm surfaces [5,6]. These observations support the need for coordinated CLA control strategies. Such strategies combine vaccination, detection, segregation or management of affected animals, sanitation, and reduction in environmental contamination.

These global features are also increasingly evident in South Korea, where CLA has become an important infectious disease constraint in Korean native goat production. Molecular studies have confirmed the circulation of *C. pseudotuberculosis* among native Korean goats and have characterized isolates recovered from skin abscesses [8,10]. Recent studies and field reports further indicate that CLA is no longer considered a sporadic disease on Korean goat farms but rather one requiring structured surveillance and prevention at the farm level [9,10,11,12]. This need is reinforced by the current status of the Korean native black goat industry, where herd expansion has been accompanied by persistent gaps in goat-specific vaccines, therapeutics, veterinary support, and routine disease surveillance [13]. Under these conditions, field-applicable vaccination strategies are needed to move CLA control from reactive lesion management toward preventive herd-health management.

Vaccination remains one of the most practical tools for reducing CLA occurrence, but the evidence base is still incomplete, particularly in goats. Previous studies using toxoid, bacterin, bacterin-toxoid, whole-cell, and recombinant antigen approaches have shown that vaccination can reduce lesion development or enhance immune responses, but protection varies according to host species, formulation, antigen composition, adjuvant, challenge model, and field condition [14,15,16,17,18,19,20,21]. Goat responses have often been less predictable than sheep responses, and field-based evidence remains comparatively limited [14,15,22]. Recent Korean studies have advanced CLA vaccine development by using local *C. pseudotuberculosis* isolates and evaluating safety, immunogenicity, and protective potential in experimental models and goats [12,22]. However, controlled experimental trials cannot fully represent the biological and management heterogeneity of commercial farms.

Another limitation of conventional CLA vaccine studies is that vaccination outcomes are usually interpreted without describing the surrounding farm microbial background. Commercial goat farms are not microbiologically uniform systems. Manure, bedding, soil, water sources, feeding areas, and animal-contact surfaces can harbor environmental bacteria, commensals, opportunistic bacteria, and other organisms relevant to farm hygiene and animal health [23,24]. These organisms are not necessarily specific to CLA, but their detection can provide information on farm-level microbial burden and potential exposure pressure. For a chronic and environmentally persistent disease such as CLA, this background is relevant when interpreting vaccine performance under natural farm conditions.

Culture-based bacteriological profiling can therefore complement CLA vaccine evaluation by placing vaccination outcomes within a farm-level microbial context. This approach does not replace pathogen-specific diagnosis of *C. pseudotuberculosis* and is not intended to infer direct causality between non-CLA organisms and vaccine outcomes. Instead, it can describe the culturable organisms present in animal-associated and environmental samples and help contextualize field observations across farms. This is particularly relevant for commercial goat farms, where CLA control depends not only on vaccination but also on hygiene, biosecurity, animal movement control, and reduction in environmental contamination.

In this study, we conducted an integrated field evaluation of a CLA vaccine in commercial goat farms and combined vaccination outcomes with farm-level bacteriological profiling. We assessed vaccine tolerability, clinical outcomes, abscess occurrence, bacterial load in abscess samples, growth performance, and CLA-specific serological responses under field conditions. We also characterized culturable organisms recovered from animal-associated and farm environmental samples. By interpreting vaccination outcomes alongside this farm-level bacteriological context, this study aimed to provide practical field evidence for CLA vaccine application and to support a broader control framework for CLA in commercial goat production.

## 2. Materials and Methods

### 2.1. Ethics Approval

The field study was conducted in client-owned commercial goat farms under veterinary supervision. All procedures involving animals were reviewed and approved by the Institutional Animal Care and Use Committee of WOOGENE B&G Co., Ltd. (Seoul, Republic of Korea) (approval number: WG-IACUC-2026-001) and were conducted in accordance with institutional guidelines and applicable national regulations for animal care and use. Written consent for animal enrollment, vaccination, clinical monitoring, blood collection, and collection of animal-associated and environmental samples was obtained from the participating farm owners before the start of the study. No experimental challenge infection, planned euthanasia, or necropsy was performed as part of this field trial.

### 2.2. Study Design

This study was designed as an integrated field evaluation of a caseous lymphadenitis (CLA) vaccine in commercial goat farms, combined with farm-level bacteriological profiling. The study included two linked components: a field vaccination trial and a bacteriological profiling survey. The vaccination trial was conducted at three commercial goat farms (Farms A, B, and C). Bacteriological profiling of animal-associated and environmental samples was performed at six commercial goat farms (Farms A–F), comprising the three vaccination-trial farms (A–C) and three additional farms (D–F) that were included only for bacteriological profiling and did not participate in the vaccination trial. The two components were analyzed in parallel: the profiling survey was intended to characterize the broader culturable microbial background of commercial goat farms and to provide descriptive context, and was not designed to explain or account for vaccine outcomes at the individual-farm level. No experimental challenge infection was performed.

The enrolled commercial goat farms were located across five provinces of South Korea (Jeollanam-do, Jeollabuk-do, Chungcheongnam-do, Gyeongsangnam-do, and Gyeongsangbuk-do in South Korea). All were conventional breeding-and-fattening operations raising Korean native black goats and Boer crossbreds, with herd sizes ranging from approximately 500 to 1000 head per farm. Animals were housed under slatted-floor or deep-litter systems depending on farm-specific conditions. Further farm-level identifying details are not disclosed to protect the confidentiality of the participating commercial farms.

The enrolled goats were approximately 2 months of age at the time of the first vaccination (based on available farm records and information provided by the farm owners) and included Korean Native Black Goats and, on some farms, New Zealand Boer crossbreds; both sexes were included. Within each farm, kidding was timed so that enrolled kids were grouped by birth date, with a maximum difference of approximately one week among individuals within the same trial group; exact individual dates of birth were not available for all animals, so minor within-group age variation (up to approximately one week) could not be entirely excluded. Because the trial was conducted on working commercial farms, the breed composition and sex distribution of the animals made available for enrollment were determined by each farm and could not be standardized by the investigators. Goats enrolled in the vaccination trial were classified according to baseline CLA antibody status before group assignment. Baseline serological status was determined using the commercial CLA ELISA kit and the in-house whole-bacterial IgG ELISA. Animals were then assigned to four field groups: seropositive vaccinated goats (SP-Vac, n = 30), seronegative vaccinated goats (SN-Vac, n = 30), seropositive non-vaccinated goats (SP-NonVac, n = 15), and seronegative non-vaccinated control goats (Control, n = 15). Vaccinated goats received two intramuscular doses of the test vaccine at week 0 and week 4, whereas non-vaccinated goats were monitored without vaccination. All animals were monitored over a 12-week (84-day) observation period following the first vaccination. Group assignment was performed to evaluate vaccine responses according to baseline serological status under natural farm exposure conditions. Because the study was conducted on working commercial farms, group sizes were determined by practical and ethical field constraints rather than by an a priori power calculation: participating farmers preferred that a larger proportion of their animals receive the vaccine, so vaccinated groups (SP-Vac and SN-Vac, n = 30 each) were larger than the corresponding non-vaccinated groups (SP-NonVac and Control, n = 15 each). This unbalanced, convenience-based assignment reflects the field nature of the study and is one reason the clinical endpoint was underpowered (Results Section 3.3).

Vaccine-associated outcomes were assessed across multiple domains: CLA-specific antibody responses were evaluated as immunogenicity; average weekly weight gain was evaluated as an indirect indicator of tolerability; and external abscess occurrence was evaluated as an exploratory clinical endpoint. Because the trial was not designed or powered to demonstrate clinical protection, the terms “vaccine efficacy” and “clinical protection” are not used to describe the present findings except where explicitly stated otherwise. Clinical signs were recorded weekly using a predefined scoring system, and blood samples were collected at scheduled time points for antibody analysis. When abscess lesions were observed, lesion samples were collected for bacteriological assessment.

**Figure 1:**
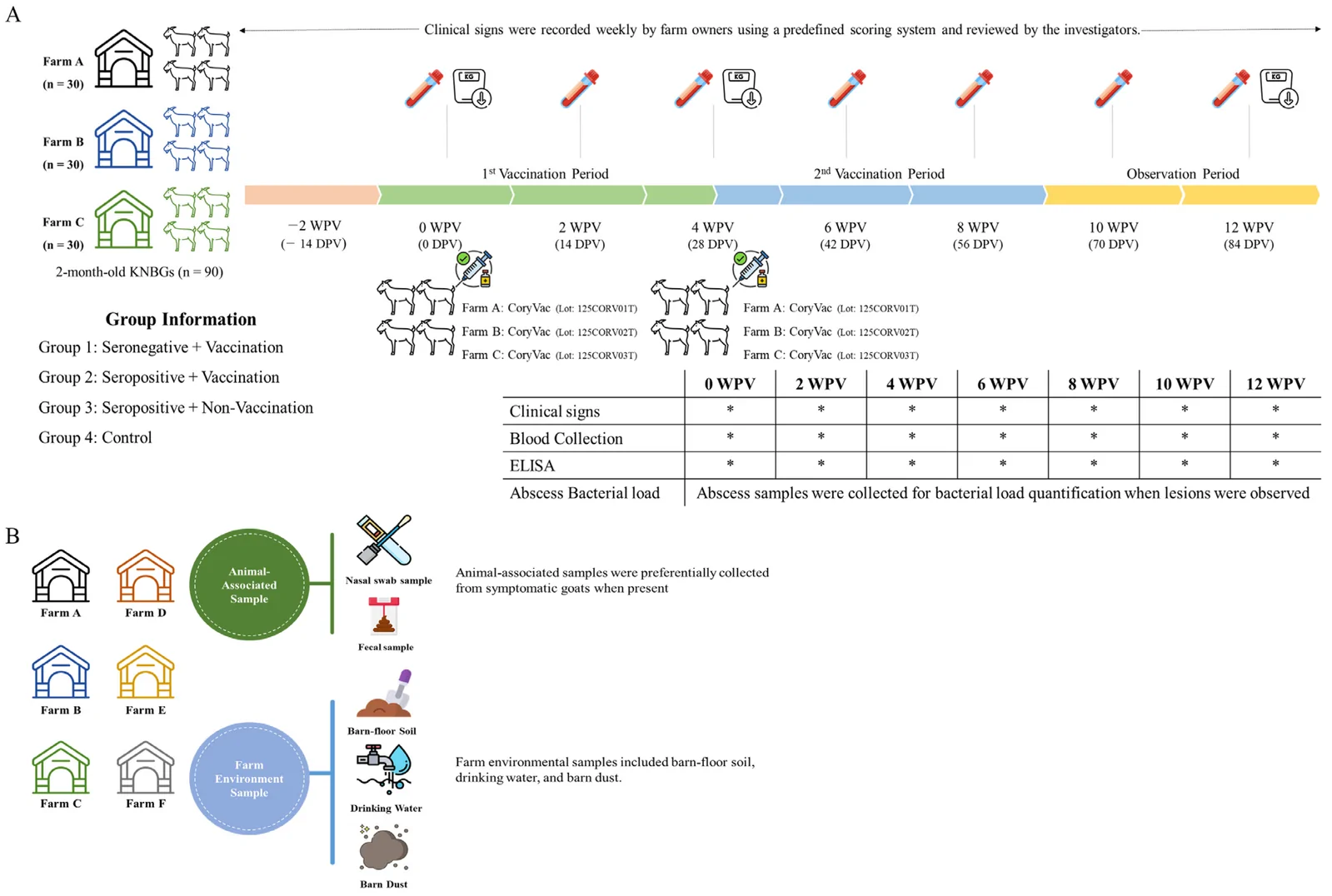
Field vaccination trial and bacteriological sampling framework for the integrated evaluation of a CLA vaccine in commercial goat farms. (**A**) Overview of the field vaccination trial conducted in commercial goat farms. Goats were classified according to baseline CLA antibody status and vaccination status into four field groups: seropositive vaccinated (SP-Vac), seronegative vaccinated (SN-Vac), seropositive non-vaccinated (SP-NonVac), and seronegative non-vaccinated control (Control) groups. Vaccinated goats received two intramuscular doses of the inactivated CLA vaccine at week 0 and week 4. Clinical monitoring, body-weight measurement, blood sampling, and abscess observation were performed during the field observation period. When external abscesses were detected, lesion material was collected for bacteriological assessment and quantification of bacterial load. (**B**) Sampling framework for animal-associated and farm environmental bacteriological profiling. Animal-associated samples included nasal swabs and rectal swabs, and environmental samples included barn-floor soil, drinking water, and barn dust. The vaccination trial (**A**) was conducted at Farms A–C, whereas bacteriological profiling (**B**) was performed at Farms A–F, with Farms D–F included for profiling only. CLA, caseous lymphadenitis; ELISA, enzyme-linked immunosorbent assay.

The bacteriological profiling component was performed to characterize the culturable bacterial background of goat farms and to provide microbiological context for field-level CLA control. Animal-associated samples included nasal swabs and fecal or rectal samples, which were preferentially collected from symptomatic goats when present. Farm environmental samples included barn-floor soil, drinking water, and barn dust. The overall animal study design and sampling framework are shown in Figure 1.

### 2.3. Test Vaccine

The test vaccine was IMMUNIS^®^ CoryVac (WOOGENE B&G., Co., Ltd., Seoul, Republic of Korea), an inactivated *Corynebacterium pseudotuberculosis* vaccine developed from a Korean Native Black Goat (KNBG) derived isolate. The selection of the vaccine strain, bacterial characterization, culture conditions, inactivation process, formulation, and experimental evaluation in animal models has been described previously [11]. Briefly, the vaccine strain (WGB-Cory) was selected from Korean goat-derived *C. pseudotuberculosis* isolates classified as biotype ovis, based on bacterial characteristics, virulence-gene profiling (including the pld gene), multilocus sequence typing, and superior growth performance, and was formulated as a formaldehyde-inactivated bacterin vaccine with carbopol and saponin as adjuvants [11].

For the present field trial, three trial vaccine lots were used: 125CORV01T, 125CORV02T, and 125CORV03T, administered at Farms A, B, and C, respectively (one lot per farm). The lots were not formally compared for immunogenicity. Each 2.0 mL field dose contained inactivated bacterial antigen equivalent to 5 × 10^6^ CFU before inactivation. The vaccine was administered by intramuscular injection into the upper shoulder region. Vaccine vials were stored at 2–8 °C, protected from light and freezing, and handled according to the manufacturer’s instructions until use.

### 2.4. Clinical Monitoring and Scoring

Clinical signs were recorded weekly by farm owners using a predefined clinical scoring sheet provided by the study team, under the supervision of the attending farm veterinarian, and the recorded observations were subsequently reviewed and compiled by the investigators. To improve consistency, the scoring criteria had been jointly reviewed and applied by the attending veterinarians and farm owners during four preliminary preclinical evaluations conducted before the field trial, so that the scoring system was interpreted consistently across observers. The scoring system was adapted from the target-animal evaluation framework used in the previous experimental study [11], in which a 0–4 scale was applied, and was modified for field monitoring by adding an intermediate severity level (ruptured abscess with depression or lethargy) to yield the present six-point scale (0–5).

Goats were assessed for general condition, external abscess formation, abscess progression, abscess rupture, depression, and mortality. Clinical signs were scored as follows: 0, clinically normal; 1, small abscess detected; 2, enlarged and hardened abscess; 3, ruptured abscess; 4, ruptured abscess with depression or lethargy; and 5, death. The number of goats developing external abscesses was recorded by farm, group, and week. When abscesses were detected, lesion material was collected for bacteriological confirmation and bacterial load quantification. Mortality, if observed, was recorded with the suspected or confirmed cause of death. Vaccinated animals were also monitored for adverse events following vaccination, including local injection-site reactions, systemic signs such as fever, and acute hypersensitivity. No vaccine-associated local or systemic adverse reactions were observed in any vaccinated goat during the study.

### 2.5. Serological Assessment of CLA-Specific Antibody Responses

Blood samples were collected at scheduled time points from baseline through the end of the observation period. Serum was separated and stored until analysis. CLA-specific antibody responses were measured using a commercial CLA ELISA kit and an in-house indirect ELISA based on inactivated whole-bacterial vaccine antigen. Antibody values were analyzed longitudinally by farm, group, individual animal, and time point. The commercial CLA ELISA kit was used as a standardized reference assay, whereas the in-house ELISA was used solely as a complementary tool for repeated longitudinal immunogenicity monitoring across farms and time points. Assay optimization (antigen coating concentration, blocking condition, and serum dilution) and comparison against the commercial CLA ELISA using paired sera were performed and are summarized in Supplementary Figure S1; however, full analytical validation, including inter- and intra-assay coefficients of variation, analytical specificity, and diagnostic sensitivity and specificity, was not performed. The in-house ELISA was therefore used for longitudinal monitoring only and is not proposed for diagnostic use.

The in-house whole-bacterial IgG ELISA was developed for this study and applied here for the first time in a vaccine field trial; it was established using an assay optimization and internal validation framework previously applied for in-house veterinary vaccine immunogenicity assays [25]. Inactivated vaccine antigen was coated onto 96-well ELISA plates at 1 × 10^4^ CFU-equivalent/well in carbonate-bicarbonate coating buffer, pH 9.6, and incubated overnight at 4 °C. Here, “CFU-equivalent” denotes the antigen amount standardized to the viable bacterial count determined before inactivation; because the coating antigen consisted of inactivated whole bacteria, this measure was used to ensure consistent antigen loading across assay batches. Post-inactivation total protein concentration was not determined. After washing four times with PBST, PBS containing 0.05% Tween 20, plates were blocked with 3% skim milk in PBS for 1 h at 37 °C. Serum samples were diluted 1:500 in conjugate diluent, PBS containing 1% skim milk, added to the plates, and incubated for 2 h at 37 °C. After washing, horseradish peroxidase-conjugated rabbit anti-goat IgG (H + L) secondary antibody (GenDEPOT, SA007-500, Katy, TX, USA), diluted 1:2000 in conjugate diluent, was added to each well and incubated for 1 h at 37 °C. Plates were washed again, developed with TMB substrate for 5 min in the dark, and the reaction was stopped with 2 M H_2_SO_4_. Optical density was measured at 450 nm using a microplate reader. For assay optimization, antigen coating concentration, blocking condition, and serum dilution were compared before application to field samples. Blank OD_450_ values were compared between 10^4^ CFU-equivalent/well and 10^5^ CFU-equivalent/well antigen coating conditions. Blocking performance was assessed using 3% and 5% skim milk in PBS, based on blank, negative-serum, and positive-serum OD_450_ values. Serum dilution was evaluated at 1:200, 1:250, and 1:500 using negative and positive goat sera. Comparative performance between the in-house whole-bacterial IgG ELISA and the commercial CLA ELISA kit was evaluated using paired serum samples.

The commercial CLA ELISA (#CK105A, HYPHEN Biomed, Neuville-sur-Oise, France) kit was used according to the manufacturer’s instructions and as previously described [11]. Final interpretation of seropositivity was based on the predefined cutoff used for each assay.

### 2.6. Quantification of Bacterial Load in Abscess Samples

When external abscess lesions were observed during the field trial, abscess material was aseptically collected for bacterial load quantification. The procedure was adapted from bacterial load quantification methods previously used for CLA-associated tissues [11,18]. Briefly, collected abscess material was weighed and homogenized or suspended in sterile phosphate-buffered saline (PBS). The homogenate was centrifuged at 2500 rpm for 10 min, and the resulting supernatant was serially diluted 10-fold in sterile PBS. Each dilution was spread onto brain heart infusion (BHI) agar plates (Difco™, Franklin Lakes, NJ, USA), and incubated aerobically at 37 °C for 48 h. After incubation, colonies at the highest dilution yielding discrete, countable colonies were counted, and representative colonies were subcultured and screened by pld-specific PCR to confirm their identity as *Corynebacterium pseudotuberculosis* (primer sequences and PCR conditions in Table 1). Bacterial load was calculated as colony-forming units (CFU) per gram of abscess material based on colonies confirmed as *C. pseudotuberculosis*.

### 2.7. Farm Environmental Sampling and Bacteriological Profiling

Farm-level bacteriological profiling was performed to characterize culturable bacteria and putative pathogenic or opportunistic bacteria present in animal-associated and environmental samples from commercial goat farms. This culture-based approach was used as a practical field-monitoring method to describe bacterial distribution by farm and sample type, rather than to establish direct causality between individual bacterial species and clinical disease.

Animal-associated samples included nasal swabs and rectal swabs. Nasal swabs were preferentially collected from goats showing respiratory signs such as nasal discharge or coughing, following the use of respiratory specimens for bacterial investigation in pneumonic goats [26]. Rectal swabs were collected from goats with diarrhea when available, as fecal or rectal sampling is routinely used for enteric bacterial monitoring in goat herds [23,27]. Barn-floor soil was collected into sterile 50 mL conical tubes, drinking water was collected into sterile tubes with the water source recorded when available, and barn dust was collected using sterile swabs. All samples were individually sealed, labeled by farm and sample type, stored at 4 °C after collection, and transported to the laboratory under refrigerated conditions.

### 2.8. Bacterial Isolation and MALDI-TOF MS Identification

Bacterial isolation was performed using culture conditions selected according to sample type. Nasal swabs and environmental samples were inoculated onto blood agar and chocolate agar plates (MB cell, Seoul, Republic of Korea). Rectal swab samples were inoculated onto eosin methylene blue agar and MacConkey agar (MB cell, Seoul, Republic of Korea) for the isolation of enteric bacteria. All plates were incubated aerobically at 37 °C for 24 h. After incubation, colony morphology was examined, and representative colonies were selected for bacterial identification.

Bacterial species were identified using matrix-assisted laser desorption/ionization-time of flight mass spectrometry (MALDI-TOF MS), a widely used method for rapid identification of cultured bacterial isolates in clinical and veterinary diagnostic microbiology [28,29,30]. Briefly, representative colonies were subjected to MALDI-TOF MS analysis using a microflex LT mass spectrometer (Bruker Daltonics, Bremen, Germany). The bacterial identification was performed by comparing the obtained mass spectra with the reference database of the instrument. Identified bacteria were summarized by farm, sample type, and presumed ecological or clinical relevance, including respiratory-associated or opportunistic bacteria, enteric bacteria, environmental or water-associated bacteria, soil-associated or spore-forming bacteria, fungal isolates, and commensal or low-priority bacteria. Samples showing fungal growth were recorded descriptively when present.

### 2.9. PCR Detection of Selected Respiratory and Enteric Pathogens

Targeted PCR assays were performed to detect selected respiratory and enteric pathogens based on farm history, clinical observations, and sample type. Nasal swab samples were tested for *Pasteurella multocida* and *Mannheimia haemolytica* as representative respiratory-associated bacterial pathogens [26,31]. Rectal swab samples were tested for bovine rotavirus and bovine coronavirus as representative enteric viral pathogens, based on previous molecular detection of these viruses in small ruminants and goat-associated diarrheal disease investigations [19,32,33].

For bacterial pathogen detection, nasal swabs were suspended in sterile PBS, and DNA was extracted using the QIAamp DNA Micro Kit (#56304, QIAGEN, Venlo, The Netherlands). For enteric viral pathogen detection, rectal swabs were suspended in sterile PBS, and nucleic acids were extracted from the clarified supernatant using the Viral Gene-spin Viral DNA/RNA Extraction Kit (iNtRON Biotechnology, Seongnam, Republic of Korea). PCR amplification was performed using pathogen-specific primers, and PCR products were analyzed by agarose gel electrophoresis. Samples were classified as positive or negative based on the presence of pathogen-specific amplicons. Primer sequences, expected amplicon sizes, and PCR conditions are provided in Table 1.

### 2.10. Statistical Analysis

Statistical analyses were performed using GraphPad Prism (ver 9.0, San Diego, CA, USA) for group-wise and farm-stratified comparisons and R software (ver 3.7.1, Vienna, Austria) for the integrated mixed-effects modeling and effect-size estimation. Baseline (pre-vaccination) values among serostatus groups were compared using the Kruskal–Wallis test. Continuous variables, including body weight, average weekly weight gain, and CLA-specific antibody responses, were summarized as mean ± standard error of the mean (SEM), unless otherwise indicated. Longitudinal antibody and growth outcomes were analyzed separately within each farm using two-way repeated-measures ANOVA, with group, time, and their interaction as fixed effects and the individual animal as the repeated unit, followed by Tukey’s multiple-comparison test (GraphPad Prism). For the integrated analysis of CLA-specific IgG trajectories across all farms, a linear mixed-effects model was fitted in R, with group, time, and their interaction as fixed effects and farm as a random effect to account for between-farm variation. Degrees of freedom for the repeated-measures and mixed-effects analyses were determined by the respective software (Prism using the standard repeated-measures partitioning, and R using the Satterthwaite approximation [34]), and are reported with the corresponding test statistics in the figure legends and Supplementary Information.

Clinical scores were summarized by group, farm, and week. Because clinical score data were ordinal and the number of abscess events was limited, clinical outcomes were primarily presented descriptively and, where appropriate, compared using non-parametric or categorical tests. Abscess occurrence, mortality, and seropositivity rates were compared among groups using Fisher’s exact test or chi-square test, depending on the number of observations per category. For the external abscess endpoint, effect estimates were additionally calculated as the risk difference (Newcombe method) [35], relative risk, and odds ratio, each with 95% confidence intervals. Because no a priori power calculation was performed for the clinical endpoint, a post hoc power analysis and a sample-size estimation were conducted for the comparison of abscess occurrence between vaccinated and non-vaccinated seropositive goats, based on the observed event rates with a two-sided α of 0.05 and using the arcsine-transformed effect size [36]. This endpoint was pre-specified as exploratory, and the trial was not powered to assess clinical efficacy.

Bacterial load in abscess samples was expressed as CFU per gram of abscess material. CFU values were log_10_-transformed before statistical comparison when sufficient quantitative data were available. Bacteriological profiling data from animal-associated and environmental samples were summarized descriptively by farm, sample type, and identified organism. These data were used to characterize farm-level microbiological context and were not interpreted as direct causal determinants of vaccine outcome. Differences were considered statistically significant at *p* < 0.05. Within each farm, CLA-specific antibody responses measured by the commercial CLA ELISA kit and the in-house whole-bacterial IgG ELISA were analyzed using two-way repeated-measures ANOVA with group, time, and their interaction as fixed effects, followed by Tukey’s post hoc test. Multiple comparisons among groups at each sampling time point were performed with Tukey’s post hoc test. Different lowercase letters above or near the data points indicate statistically significant differences among groups at the same time point (*p* < 0.05).

**Table 1:** Primer Sequences, expected amplicon sizes, and PCR conditions.

Target Organism/Pathogen	Gene	Amplicon Size (bp)	Primer Sequences (5′ to 3′)	Ref.
*Corynebacterium* *pseudotuberculosis*	PLD	924	F: ATGAGGGAGAAAGTTGTTTTA R: TCACCACGGGTTATCCGC	[13]
	1 Cycle: Initial Denaturation (94 °C, 5 min), 30 Cycles: Denaturation (94 °C, 30 s)–Annealing (54 °C, 30 s)–Extension (72 °C, 90 s), 1 Cycle: Final Extension (72 °C, 10 min)			
*Pasteurella* *multocida*	KMT1	460	F: ATCCGCTATTTACCCAGTGG R: GCTGTAAACGAACTCGCCAC	[37]
	1 Cycle: Initial Denaturation (95 °C, 5 min), 30 Cycles: Denaturation (95 °C, 30 s)–Annealing (55 °C, 30 s)–Extension (72 °C, 30 s), 1 Cycle: Final Extension (72 °C, 5 min)			
*Mannheimia* *haemolytica*	lktA-artJ intergenic region	385	F: GTCCCTGTGTTTTCATTATAAG R: CACTCGATAATTATTCTAAATTAG	[38]
	1 Cycle: Initial Denaturation (95 °C, 3 min), 35 Cycles: Denaturation (94 °C, 1 min)–Annealing (58 °C, 1 min)–Extension (72 °C, 1 min), 1 Cycle: Final Extension (72 °C, 1 min)			
Bovine rotavirus	VP7	1062	F: GCCTTTAAAAGCGAGAATTT R: GGTCACATCATACAAYTCTA	[39]
	1 Cycle: Initial Denaturation (94 °C, 5 min), 30 Cycles: Denaturation (94 °C, 1 min)–Annealing (56 °C, 1 min)–Extension (72 °C, 2 min), 1 Cycle: Final Extension (72 °C, 8 min)			
Bovine coronavirus	HE	660	F: GCACCACTTCTGGTAGTAATGA R: CAGCATCCCCATGATTGATATC	Intron Biotechnology Co., Ltd. (Seongnam, Republic of Korea) “KR101064866B1”
	Reverse transcription: (45 °C, 30 min), 1 Cycle: Initial Denaturation (95 °C, 5 min), 40 Cycles: Denaturation (95 °C, 30 s)–Annealing (58 °C, 1 min)–Extension (72 °C, 2 min), 1 Cycle: Final Extension (72 °C, 5 min)			

Abbreviations: bp, base pairs; F, forward primer; R, reverse primer; PCR, polymerase chain reaction. Primer sequences are shown in the 5′ to 3′ orientation. The degenerate nucleotide Y indicates C or T according to the IUPAC nucleotide code. Amplicon sizes and PCR conditions were based on the cited references or patent source and were used for endpoint detection by agarose gel electrophoresis. For bovine rotavirus and bovine coronavirus, viral RNA was reverse-transcribed before PCR amplification. The bovine coronavirus primer set targets the hemagglutinin esterase (HE) gene and was derived from Korean Patent KR101064866B1.

For the integrated analysis, CLA-specific in-house IgG values were summarized by treatment group and by days post-vaccination (DPV) using R. Group trajectories were plotted as mean ± SEM at each time point. Individual fold changes were calculated as the ratio of IgG values at DPV 56 to the corresponding values at DPV −14, and group-level fold-change values were then calculated from individual animal ratios. Abscess-associated events were overlaid on the group-level IgG trajectories using the corresponding individual animal IgG value at the time of abscess detection. Bacterial loads from abscess samples were log10-transformed for graphical scaling of event markers, and clinical scores at abscess detection were displayed as categorical marker fills. Abscess incidence between SP-Vac and SP-NonVac animals was compared using Fisher’s exact test, and differences in IgG levels between SP-Vac and SP-NonVac animals over the indicated post-vaccination period were evaluated using the Mann–Whitney U test.

For Supplementary Figure S1, assay optimization data were summarized as mean ± SD. Positive-to-negative ratios and OD_450_ differences between positive and negative sera were calculated to support selection of blocking and serum dilution conditions. Agreement between the in-house ELISA and the commercial CLA ELISA was evaluated using Spearman’s correlation, Pearson’s correlation, linear regression, cutoff-based classification agreement, and Bland–Altman-style difference analysis. A cutoff of 0.40 was applied to both readouts for the classification agreement analysis unless otherwise indicated.

## 3. Results

### 3.1. Baseline Serostatus-Based Group Assignment and Growth Performance Under Field Conditions

**Figure 2:**
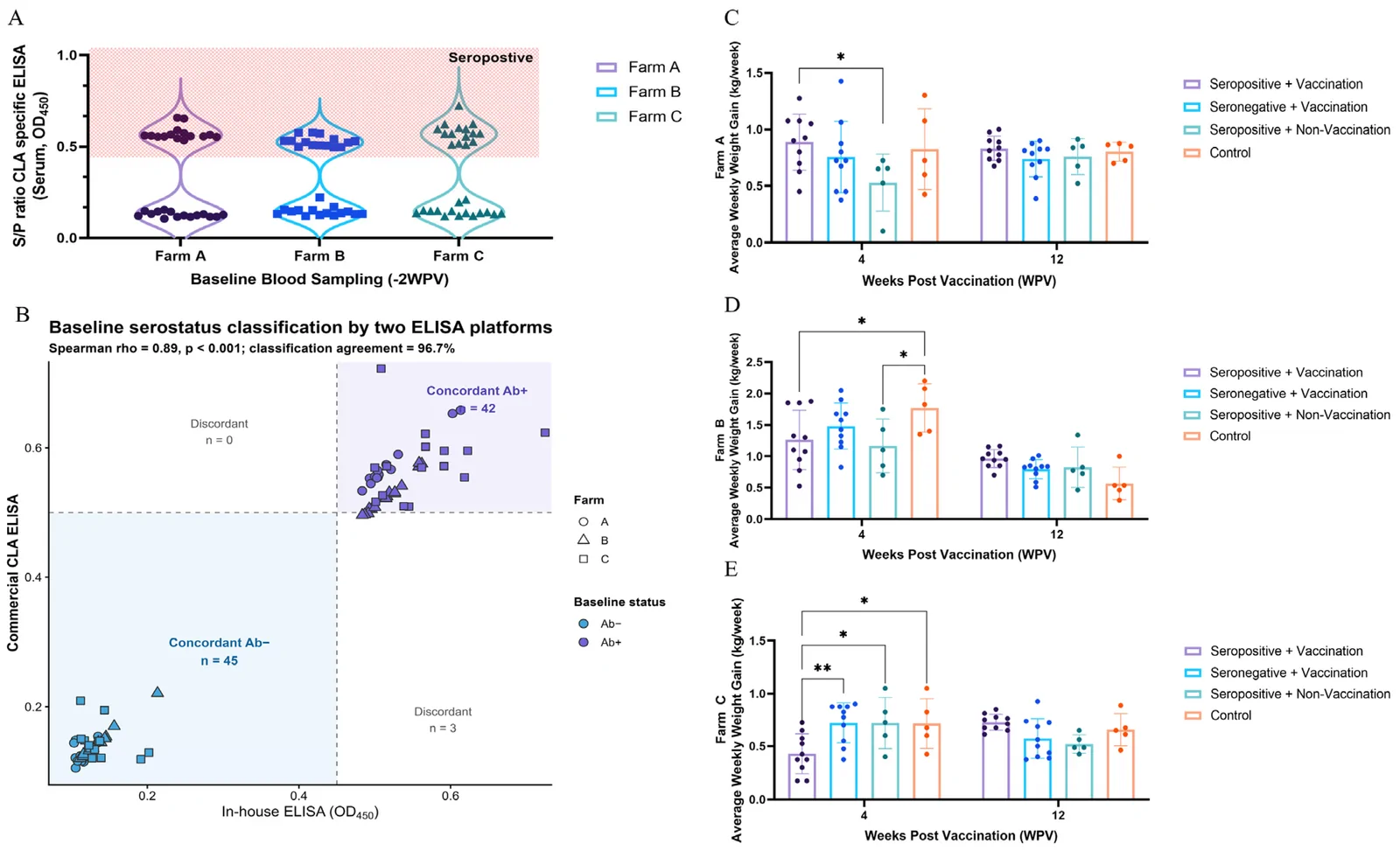
Baseline serostatus classification and growth performance after field vaccination. (**A**) Baseline CLA-specific antibody status of goats before vaccination, showing separation between seronegative and seropositive animals at −2 weeks post-vaccination (WPV). (**B**) Concordance between the commercial CLA ELISA kit and the in-house whole-bacterial IgG ELISA for baseline serostatus classification. Spearman’s correlation coefficient and classification agreement are shown in the panel. (**C**–**E**) Farm-specific average weekly weight gain at 4 and 12 WPV in Farm A (**C**), Farm B (**D**), and Farm C (**E**). Individual points represent individual goats, and bars indicate mean ± SEM. Asterisks indicate statistically significant differences between groups (* *p* < 0.05, ** *p* < 0.01). CLA, caseous lymphadenitis; ELISA, enzyme-linked immunosorbent assay; SEM, standard error of the mean; WPV, weeks post-vaccination.

Baseline CLA-specific antibody levels were assessed before vaccination to support serostatus-based group assignment. At baseline, 2 weeks before vaccination, goats from the three farms showed a clear separation between baseline seronegative and seropositive populations, indicating pre-existing variation in CLA antibody status under field conditions (Figure 2A). To evaluate the consistency of baseline classification, antibody values measured by the in-house ELISA were compared with those obtained using a commercial CLA ELISA kit. The two assays showed strong concordance, with a Spearman’s correlation coefficient of 0.89 (*p* < 0.001) and a classification agreement of 96.7% (Figure 2B). Most animals were consistently classified as antibody-negative or antibody-positive by both assays, supporting baseline serostatus-based group assignment. Additional optimization and preliminary comparative assessment of the in-house whole-bacterial IgG ELISA are provided in Supplementary Figure S1. In the assay optimization step, the 1 × 10^4^ CFU-equivalent/well antigen coating condition showed a lower blank OD_450_ than the 1 × 10^5^ CFU-equivalent/well condition and was close to the commercial kit blank level. The 3% skim milk blocking condition produced lower blank and negative-serum background than the 5% skim milk condition, while maintaining a clear positive-serum signal. Serum dilution testing showed that 1:500 preserved positive-negative separation while reducing negative-serum background. In paired sample analysis, the in-house ELISA showed strong agreement with the commercial CLA ELISA across 90 samples, with Spearman’s rho = 0.980, Pearson r = 0.995, R^2^ = 0.990, and 85/90 classification agreement (94.4%) when a cutoff of 0.40 was applied to both readouts.

Growth performance was evaluated as a field tolerability outcome after vaccination. As the enrolled goats were young, growing animals (approximately 2 months of age at first vaccination), average weekly weight gain reflects normal growth during the rearing period. Average weekly weight gain showed farm-dependent variation at 4 WPV, with several significant pairwise differences observed within individual farms (Figure 2C–E). However, these differences were not consistently maintained at 12 WPV. Across farms, vaccinated goats did not show a persistent reduction in weight gain compared with the corresponding non-vaccinated groups. These results indicate that vaccination was not associated with an evident growth penalty under commercial farm conditions, although growth responses varied among farms.

### 3.2. Field Vaccination Induces CLA-Specific Antibody Responses in Goats

**Figure 3:**
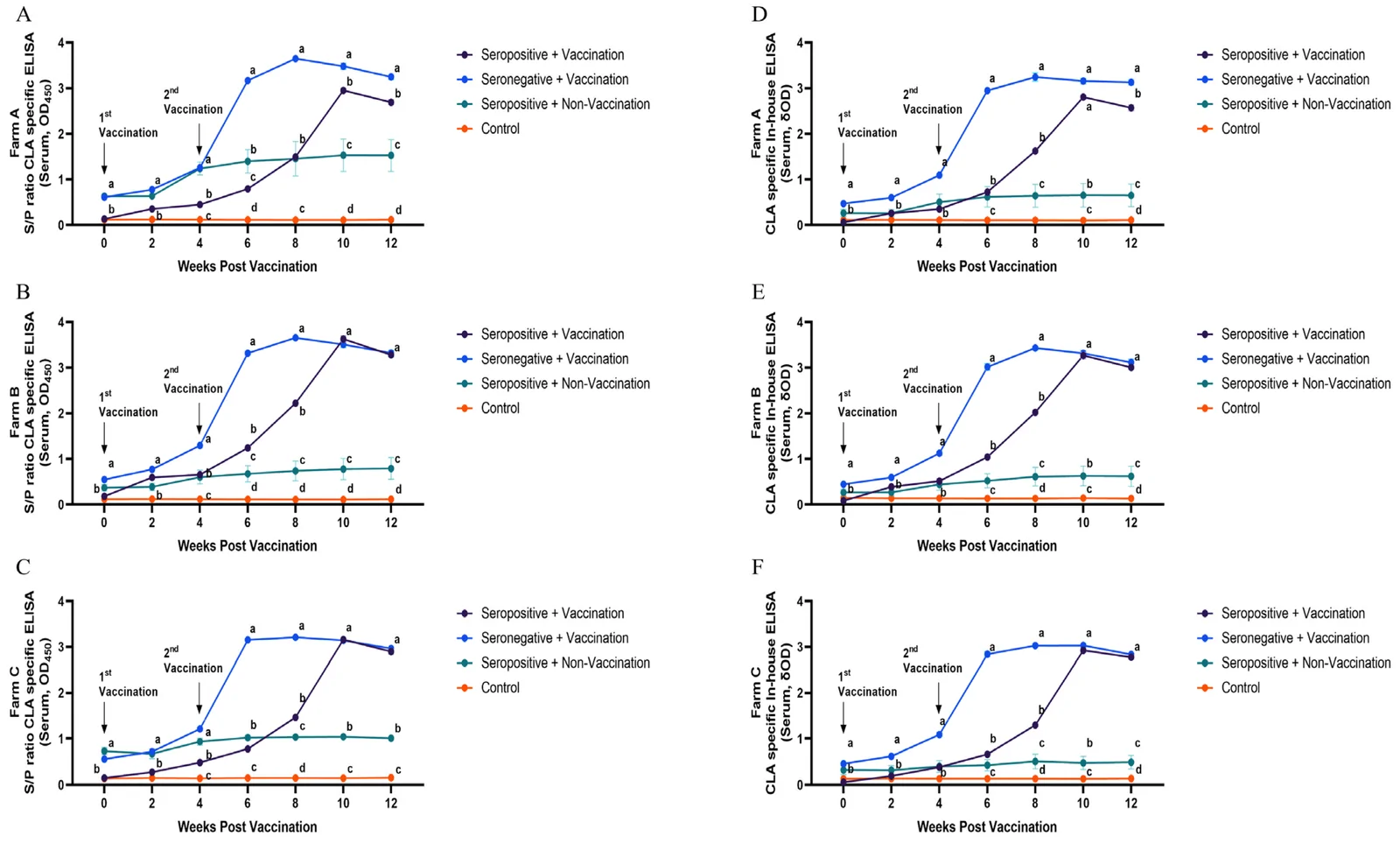
CLA-specific antibody responses in vaccinated and non-vaccinated goats under field conditions. (**A**–**C**) Serum antibody responses measured using a commercial CLA ELISA kit in Farm A (**A**), Farm B (**B**), and Farm C (**C**). (**D**–**F**) CLA-specific IgG responses measured using an in-house whole-bacterial IgG ELISA in Farm A (**D**), Farm B (**E**), and Farm C (**F**). Goats were grouped according to baseline CLA antibody status and vaccination status as seropositive vaccinated (SP-Vac), seronegative vaccinated (SN-Vac), seropositive non-vaccinated (SP-NonVac), and seronegative non-vaccinated control (Control) groups. Vaccinated goats received the first vaccination at week 0 and the second vaccination at week 4. Data are presented as mean ± SEM. Different lowercase letters indicate statistically significant differences among groups at the same time point within each farm (*p* < 0.05). CLA, caseous lymphadenitis; ELISA, enzyme-linked immunosorbent assay; IgG, immunoglobulin G; OD_450_, optical density at 450 nm; S/P, sample-to-positive ratio; SEM, standard error of the mean.

CLA-specific antibody responses were evaluated using a commercial CLA ELISA kit and an in-house whole-bacterial IgG ELISA (Figure 3). In the commercial ELISA analysis, vaccinated goats from all three farms showed increased serum S/P ratios after vaccination, whereas non-vaccinated control animals remained consistently low throughout the observation period (Figure 3A–C). Among goats that were seronegative before vaccination, antibody responses increased markedly after booster vaccination and remained elevated until 12 weeks post-vaccination. Goats that were seropositive before vaccination also showed increased antibody responses over time, although the increase was more gradual and varied among farms. In contrast, seropositive, non-vaccinated goats maintained lower, relatively stable antibody levels.

Likewise, the in-house whole-bacterial IgG ELISA showed a similar vaccine-associated response pattern across the three farms (Figure 3D–F). Seronegative vaccinated goats showed a rapid increase in CLA-specific IgG after booster vaccination, followed by sustained high antibody levels through the end of the observation period. Seropositive vaccinated goats also showed progressive increases in IgG responses, with peak responses generally occurring later than those observed in seronegative vaccinated goats. Non-vaccinated seropositive goats remained at lower antibody levels, and control animals showed minimal responses throughout the study. Together, the commercial CLA ELISA and the in-house IgG ELISA showed consistent vaccine-associated response patterns under commercial farm conditions. Because the two assays report results on different scales (S/P ratio for the commercial assay and OD_450_ for the in-house assay), they were compared only in terms of the direction and time course of the antibody response and not by direct numerical comparison of their values.

### 3.3. Abscess Occurrence and CLA-Specific IgG Trajectories According to Baseline Serostatus and Vaccination Status

**Figure 4:**
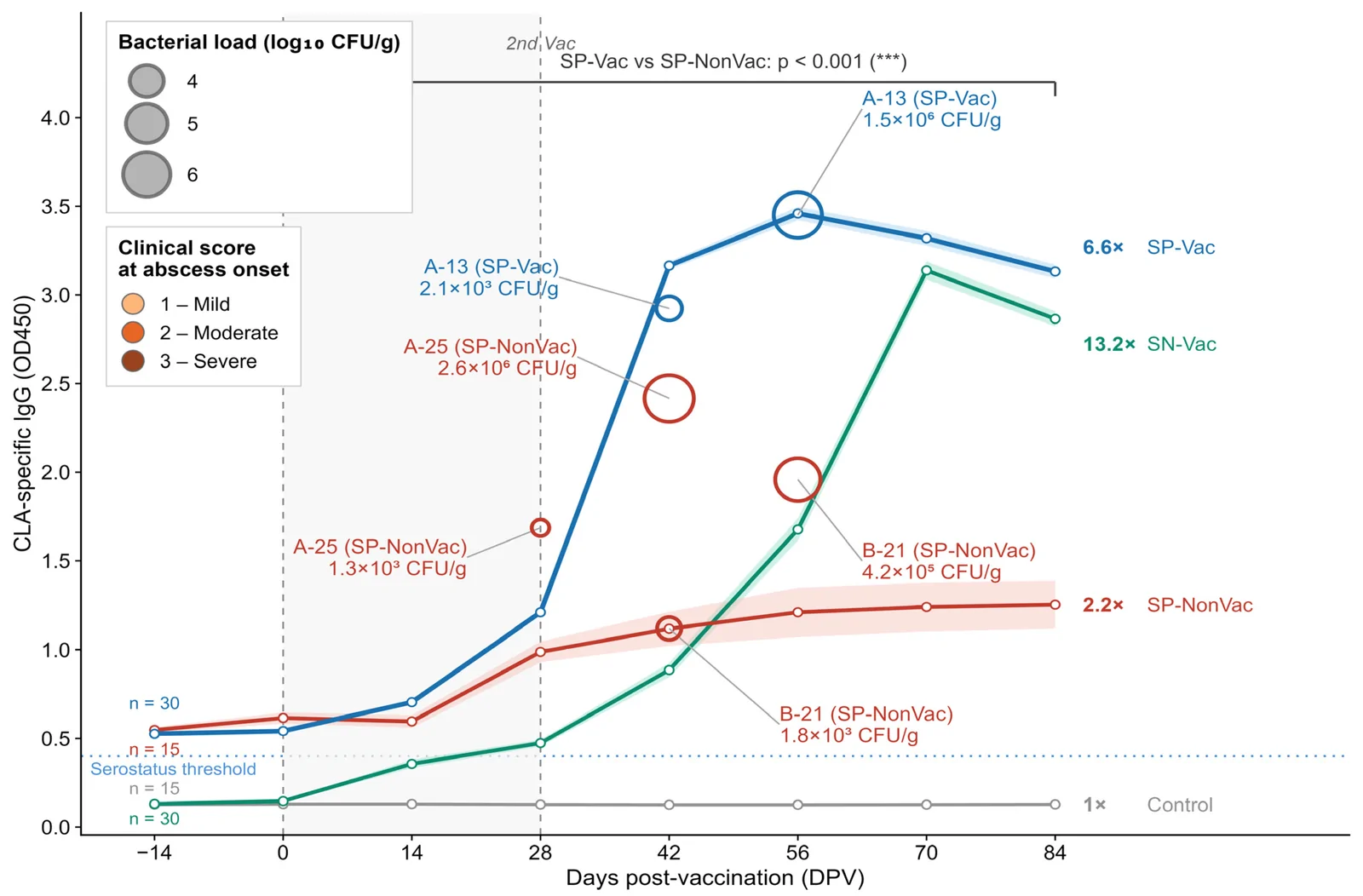
Integrated analysis of CLA-specific IgG trajectories, abscess occurrence, and abscess bacterial loads according to baseline serostatus and vaccination status. CLA-specific IgG responses measured by the in-house whole-bacterial ELISA were plotted longitudinally from DPV −14 to DPV 84 in four groups: seropositive vaccinated (SP-Vac, *n* = 30), seronegative vaccinated (SN-Vac, *n* = 30), seropositive non-vaccinated (SP-NonVac, *n* = 15), and seronegative non-vaccinated control (Control, *n* = 15) goats. Lines indicate group means, and shaded ribbons indicate mean ± SEM. The horizontal dotted line indicates the serostatus threshold used for baseline group assignment. Vertical dashed lines indicate the timing of the first vaccination at DPV 0 and the second vaccination at DPV 28, and the shaded interval indicates the period between the two vaccinations. Fold-change labels on the right side indicate the mean group-level IgG fold increase from DPV −14 to DPV 56. The significance bracket indicates the comparison of CLA-specific IgG levels (immunogenicity) between SP-Vac and SP-NonVac goats over the indicated post-vaccination period. Circular event markers indicate individual abscess-associated samples with quantified bacterial loads. Marker size represents bacterial load on a log10 CFU/g scale, marker fill indicates the clinical score at abscess detection, and the outer ring color indicates the group of the affected animal, with blue denoting SP-Vac and red denoting SP-NonVac. Labels indicate animal identity, group, sampling time point, and bacterial load. *** indicates *p* < 0.001 for the comparison between SP-Vac and SP-NonVac. CLA, caseous lymphadenitis; CFU, colony-forming units; DPV, days post-vaccination; ELISA, enzyme-linked immunosorbent assay; IgG, immunoglobulin G; SEM, standard error of the mean.

Abscess occurrence was recorded in relation to baseline serostatus and vaccination status during the observation period (Figure 4). Among 45 seronegative animals, including SN-Vac (*n* = 30) and Control (*n* = 15), no external abscess formation was observed. In contrast, 3 of 45 seropositive animals (6.7%) developed external abscesses with quantifiable bacterial loads. These cases occurred on two farms, Farms A and B.

Abscess cases were considered confirmed when *C. pseudotuberculosis* was identified from the lesion material by pld-specific PCR, distinguishing CLA abscesses from skin lesions of other etiology. Within the seropositive groups, 1 of 30 SP-Vac animals (3.3%) and 2 of 15 SP-NonVac animals (13.3%) developed abscesses. The difference in abscess incidence between SP-Vac and SP-NonVac animals was not statistically significant (Fisher’s exact test, *p* = 0.254; risk difference −10.0%, 95% CI −34.7% to 6.4%; relative risk 0.25, 95% CI 0.02 to 2.54; odds ratio 0.27, 95% CI 0.03 to 2.30). All confidence intervals included the null value. A post hoc analysis indicated that, given the observed event rates and group sizes, the study had less than 30% power to detect a difference of this magnitude at a two-sided α of 0.05, and that approximately 109 animals per group would be required to achieve 80% power. This clinical endpoint was therefore underpowered, and the non-significant difference should not be interpreted as evidence of either the presence or the absence of clinical protection.

CLA-specific IgG levels were measured longitudinally from DPV −14 to DPV 84 (Figure 4). SP-Vac and SP-NonVac animals showed comparable baseline IgG levels before vaccination. After vaccination, SP-Vac animals showed higher IgG levels than SP-NonVac animals from DPV 14 onward (Mann–Whitney U test, *p* < 0.001), and this difference was maintained through DPV 84. This significant difference reflects a stronger vaccine-induced antibody response (immunogenicity) in vaccinated goats. It should not be interpreted as evidence of clinical protection, which was not demonstrated in this study. At DPV 56, the mean IgG fold increase from baseline was 6.6-fold in SP-Vac animals and 2.2-fold in SP-NonVac animals. SN-Vac animals showed a 13.2-fold increase from baseline, whereas Control animals showed a 1.0-fold change.

The three confirmed abscess cases were overlaid on the group IgG trajectories. Animal A-13 in the SP-Vac group developed an abscess at DPV 42, with a bacterial load of 2.1 × 10^3^ CFU/g, followed by 1.5 × 10^6^ CFU/g at DPV 56. The clinical score at DPV 56 was 3. In the SP-NonVac group, animal A-25 developed an abscess at DPV 28, with a bacterial load of 1.3 × 10^3^ CFU/g, followed by 2.6 × 10^6^ CFU/g at DPV 42. Animal B-21 developed an abscess at DPV 42, with a bacterial load of 1.8 × 10^3^ CFU/g, followed by 4.2 × 10^5^ CFU/g at DPV 56. These event-level data summarize the timing, clinical score, and bacterial burden of confirmed abscess cases in relation to group-level IgG trajectories.

### 3.4. Culture and MALDI-TOF MS Identification Revealed Farm-Dependent Culturable Isolate Profiles

**Figure 5:**
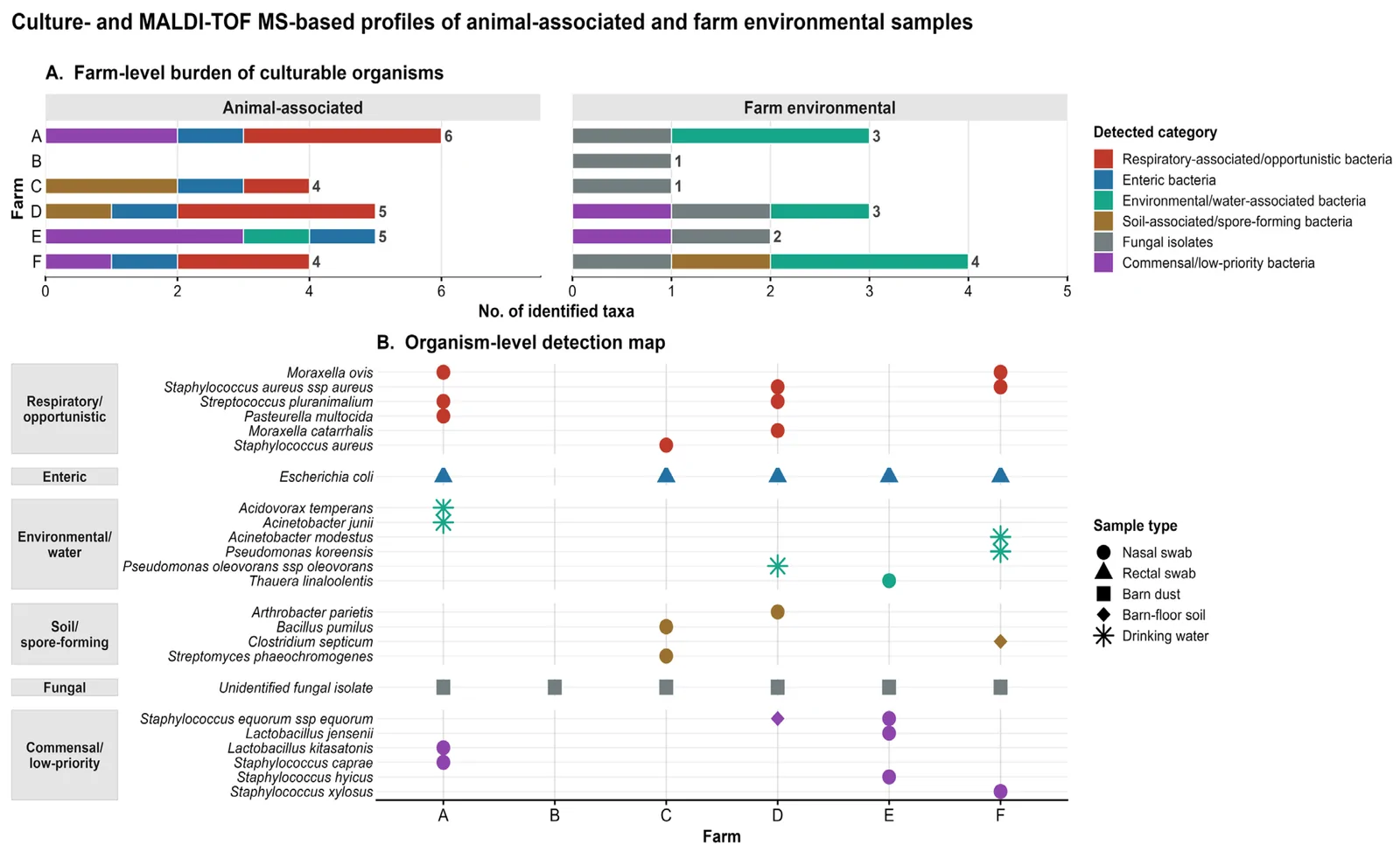
Culture- and MALDI-TOF MS-based profiles of animal-associated and farm environmental samples. Profiling was performed at six farms (Farms A–F); Farms A–C corresponded to the vaccination-trial farms, and Farms D–F were included for bacteriological profiling only. (**A**) Farm-level burden of culturable organisms identified from animal-associated and farm environmental samples. Bars indicate the number of distinct organisms recovered by culture and identified by MALDI-TOF MS in each farm and sample-source category. Numbers at the end of bars indicate the total number of identified organisms per farm and source category. (**B**) Organism-level detection map showing the distribution of identified isolates across farms and sample types. Each point indicates the detection of a given organism in the corresponding farm and sample type, with colors representing predefined microbiological categories and symbols representing sample types. Identified organisms were grouped into respiratory-associated or opportunistic bacteria, enteric bacteria, environmental or water-associated bacteria, soil-associated or spore-forming bacteria, fungal isolates, and commensal or low-priority bacteria. This profiling was performed descriptively to characterize the culturable microbial background of commercial goat farms and was not used to establish causality between individual organisms and vaccine outcomes. MALDI-TOF MS, matrix-assisted laser desorption/ionization-time of flight mass spectrometry.

Bacteriological profiling was performed at six farms (Farms A–F), of which Farms A–C were the vaccination-trial farms and Farms D–F were included for profiling only. Culture followed by MALDI-TOF MS identification revealed farm-dependent differences in culturable isolate profiles across these farms (Figure 5). In animal-associated samples, identified taxa were detected in five of six farms, with four to six taxa detected among positive farms (Figure 5A). Farm A showed the highest animal-associated isolate burden, whereas Farm B had no identified taxa in animal-associated samples under the present culture conditions. Farm environmental samples yielded identified taxa in all six farms, with one to four taxa detected per farm. Environmental isolate burden was highest in Farm F, followed by Farms A and D.

The organism-level detection map showed that animal-associated isolates were mainly respiratory-associated or opportunistic bacteria (e.g., *Pasteurella multocida*, *Moraxella* spp., and *Staphylococcus* spp.) and enteric bacteria, with *Escherichia coli* the most consistently detected enteric organism, whereas environmental samples contributed more strongly to environmental or water-associated bacteria, soil-associated or spore-forming bacteria (including *Clostridium septicum*), and fungal isolates (Figure 5B). The full list of identified taxa by category, farm, and sample type is provided in Figure 5B and Table 2.

Together, these culture- and MALDI-TOF MS-based results show that the field farms differed not only in the number of culturable taxa but also in the types of organisms recovered from animal-associated and environmental samples. This profiling was not intended to establish causality between specific organisms and vaccine outcomes but provided microbiological context for interpreting vaccine performance under natural commercial farm conditions.

### 3.5. Selected Respiratory, Enteric, and Environmental Organisms Showed Farm-Dependent Detection Patterns

Selected respiratory, enteric, and environmental organisms were summarized by farm to identify organisms relevant to farm-level monitoring (Table 2). Among nasal swab samples, *Pasteurella multocida* and *Mannheimia haemolytica* were detected by targeted PCR in 4 of 22 and 7 of 22 samples, respectively. At the farm level, *P. multocida* was detected in Farms A, D, and E, whereas *M. haemolytica* was detected in Farms A, B, D, and F. In addition to these PCR targets, culture followed by MALDI-TOF MS identified respiratory-associated or opportunistic bacteria, including *Streptococcus pluranimalium* in Farms A and D and *Staphylococcus aureus* in Farms C, D, and F. Notably, *C. pseudotuberculosis*, the causative agent of CLA, was not recovered from any animal-associated or environmental sample under the culture conditions used, as the profiling was a general culture and MALDI-TOF MS survey rather than a targeted assay for this fastidious pathogen.

Enteric viral pathogens also showed farm-dependent detection. Bovine rotavirus was detected by PCR in 4 of 19 rectal swab samples in Farms A, B, and C. Bovine coronavirus was detected in 9 of 19 rectal swab samples in Farms A, B, C, and F. Among environmental samples, *Clostridium septicum* was identified from barn-floor soil in Farm F. Overall, these findings indicate that selected organisms associated with respiratory, enteric, or environmental monitoring were not uniformly distributed across farms. This table complements the broader culture- and MALDI-TOF MS-based isolate profiling by highlighting organisms with practical relevance for farm-level disease monitoring.

## 4. Discussion

This field study extends the previous experimental evaluation of a Korean inactivated CLA vaccine into commercial goat farm conditions [11]. The previous work established the vaccine strain, inactivation-based formulation, safety profile, immunogenicity, and protective potential under controlled experimental settings [11]. The present study evaluated the same vaccine concept under natural farm exposure, where baseline antibody status, farm management, microbial background, and clinical event timing cannot be experimentally standardized. This field setting is directly relevant to CLA control because CLA is a chronic herd-level disease in which clinically visible abscesses represent only part of the infection process [1,2,3,4,20,40]. Therefore, vaccine performance under commercial conditions should be interpreted using multiple outcome domains, including serology, clinical monitoring, abscess occurrence, bacterial load, growth performance, and farm-level bacteriological context.

Baseline serological status was a key factor in this field population. CLA can persist in herds through subclinical infection, delayed external abscess formation, rupture of mature lesions, environmental contamination, and repeated exposure of susceptible animals [1,2,3,4,5,6,20,40]. These persistence mechanisms are drawn from previous literature; the present study did not measure the environmental survival or persistence of *C. pseudotuberculosis*, and this factor is discussed only as general background rather than as a study finding. Experimental infection data in goats have also shown that gross lesions can appear before serological detection becomes reliably positive, indicating that clinical stage and antibody status may not fully overlap during the early course of infection [41]. In the present study, baseline group assignment was supported by strong concordance between the commercial CLA ELISA and the in-house whole-bacterial IgG ELISA, with a Spearman’s rho of 0.89 and 96.7% classification agreement. This supports the use of baseline antibody status as a practical stratification variable in field vaccine studies. However, seropositivity should not be interpreted as equivalent to protective immunity because CLA serological assays differ according to antigen format, host species, assay design, cutoff definition, and diagnostic purpose [20,21,42,43,44,45,46,47,48,49].

The in-house whole-bacterial IgG ELISA was included to support repeated longitudinal monitoring across farms and time points. The commercial CLA ELISA kit provided a standardized reference assay, but repeated field testing can be limited by kit availability, lot access, and cost. Therefore, the in-house ELISA was used as a complementary immunogenicity-monitoring assay rather than as a replacement diagnostic kit. This approach followed the assay-development logic previously applied for in-house veterinary vaccine immunogenicity assessment, where assay optimization and internal validation were used to support repeated serological monitoring [25]. The use of whole-bacterial, culture-supernatant, or sonicated *C. pseudotuberculosis* antigens in ELISA is also consistent with previous CLA serological studies [42,43,44,45,46,47,48,49,50]. In this study, the in-house ELISA showed longitudinal response patterns that were consistent with the commercial CLA ELISA, including increased antibody responses in vaccinated goats and low responses in non-vaccinated controls. These findings support its use for within-study longitudinal monitoring, while further analytical validation would be required before independent diagnostic application.

Vaccination induced consistently detectable CLA-specific antibody responses across the three farms. In both the commercial CLA ELISA and the in-house whole-bacterial IgG ELISA, seronegative vaccinated goats showed marked antibody increases after booster vaccination, whereas seropositive vaccinated goats showed a more gradual increase. This pattern is consistent with differences in baseline immune history. Animals classified as seronegative at enrollment had low initial antibody levels and therefore showed larger fold increases after vaccination, whereas seropositive animals already had detectable antibody levels before the first dose. Previous CLA vaccine studies have shown that toxoid, bacterin, bacterin-toxoid, whole-cell, and recombinant antigen approaches can induce humoral responses, although the magnitude and clinical relevance of these responses vary by host species, antigen composition, adjuvant, challenge model, and field condition [14,15,16,17,18,21,51]. Thus, the antibody responses observed here support vaccine immunogenicity under commercial farm conditions, but they should not be interpreted as a complete correlate of protection.

The abscess outcome provides a clinically relevant but statistically limited field endpoint. External abscesses were detected in 3 of 45 baseline seropositive goats, whereas none were detected among 45 baseline seronegative goats. Within the baseline seropositive population, abscesses occurred in 1 of 30 vaccinated goats and 2 of 15 non-vaccinated goats, corresponding to 3.3% and 13.3%, respectively. This difference was not statistically significant by Fisher’s exact test (*p* = 0.254), and therefore does not demonstrate statistically confirmed clinical protection. Previous CLA vaccine studies have reported reduced lesion development or lower clinical occurrence in vaccinated animals under experimental or field conditions [14,15,16,17,18,21]; however, the present field study was not designed or powered to test this outcome. The difference in external abscess occurrence between vaccinated and non-vaccinated seropositive goats was not statistically significant, and all effect estimates were accompanied by wide confidence intervals that included the null value (Results Section 3.3). Because of the very small number of abscess events, this comparison was underpowered and does not permit any conclusion about clinical protection in either direction: the non-significant result should not be interpreted as evidence of vaccine efficacy, nor as evidence of vaccine non-efficacy (i.e., it reflects a Type II error risk). These findings should therefore be interpreted solely as field evidence of vaccine-induced immunogenicity and tolerability, with the external abscess endpoint regarded strictly as exploratory and hypothesis-generating rather than as a demonstration of clinical protection.

The limited number of abscess cases also reflects the difficulty of evaluating CLA vaccines under commercial farm conditions. Experimental challenge models allow control of dose, route, timing, and lesion assessment, but they do not reproduce the full heterogeneity of natural farm exposure [11,18,51]. Field studies capture real management and exposure conditions, but infection pressure, exposure timing, and the probability of observing external abscesses cannot be standardized [21,37]. In this study, clinical monitoring focused on externally detectable abscesses, and internal lesions were not assessed by necropsy or imaging. Because CLA frequently presents as internal abscesses that cannot be detected externally, reliance on external abscess detection may underestimate the true disease burden, and vaccinated animals could have developed internal lesions while appearing clinically normal. Consequently, the absence or low frequency of external abscesses during the observation period cannot be interpreted as evidence of clinical protection of any kind [1,2,3,20,41]. The clinical data are therefore best interpreted as event-based field observations linked to baseline serostatus, vaccination status, IgG kinetics, clinical score, and bacterial load.

The interpretation of antibody responses should also consider the biology of *C. pseudotuberculosis*. This organism is a facultative intracellular, Gram-positive bacterium whose pathogenesis involves tissue invasion, survival within host cells, lymphatic dissemination, and abscess formation, with phospholipase D acting as a major virulence factor [2,4,7,8,20,51]. Antibody responses are useful indicators of vaccine inoculations and exposure history; however, because *C. pseudotuberculosis* is a facultative intracellular pathogen, humoral responses alone cannot establish protective immunity or a plausible immune correlate of protection [46,47,48]. Cell-mediated responses, including antigen-specific T-cell activation, IFN-γ production, and macrophage-associated effector functions, are considered central to protection against intracellular pathogens but were not assessed in the present study [16,18]. The IgG responses reported here should therefore be interpreted as evidence of vaccine-induced antibody induction rather than as a demonstration of protective immunity. Accordingly, the immunological mechanisms underlying potential vaccine-associated protection remain to be clarified in future studies incorporating cellular immune assessment. Future field studies should therefore include cellular immune readouts, longer clinical follow-up, lesion-level pathogen confirmation, and assessment of internal lesions when feasible. Such endpoints would help clarify whether vaccine-induced responses are associated with reduced abscess frequency, delayed lesion progression, lower bacterial burden, or reduced environmental contamination risk.

Growth performance was included as a field tolerability outcome. Across farms, vaccinated goats did not show a persistent reduction in average weekly weight gain compared with corresponding non-vaccinated groups. This observation is relevant because a field vaccine must be acceptable in terms of animal performance and farm management, not only in terms of serological response. Early farm-dependent differences in weight gain were observed, but these differences were not consistently maintained at 12 WPV. Because growth in these young goats (approximately 2 months of age) can be affected by nutrition, baseline body condition, stocking density, concurrent disease pressure, and management practices, average weekly weight gain should be interpreted as a tolerability readout rather than as a measure of clinical protection or efficacy. This supports the use of multiple outcome domains when evaluating CLA vaccination under commercial farm conditions [1,2,3,18,40].

The culture- and MALDI-TOF MS-based profiling results showed that the enrolled farms differed in both the number and type of culturable taxa recovered from animal-associated and environmental samples. Animal-associated samples mainly yielded respiratory-associated or opportunistic bacteria and enteric bacteria, whereas environmental samples contributed more strongly to environmental or water-associated bacteria, soil-associated or spore-forming bacteria, and fungal isolates. Among the spore-forming organisms, *Clostridium septicum* was recovered from barn-floor soil on one farm (Farm F). Although unrelated to CLA, *C. septicum* is a soil-associated pathogen and a recognized cause of gas gangrene (malignant edema) in ruminants, including goats [52]; its detection illustrates the type of environmental pathogen pressure that can coexist with CLA on commercial goat farms and that may be relevant to overall farm-level biosecurity. MALDI-TOF MS is widely used for rapid identification of cultured bacterial isolates in clinical and veterinary diagnostic microbiology [28,29,30]. In this study, MALDI-TOF MS was not used to define the complete microbiome and was not intended to establish causality between non-CLA organisms and vaccine outcome. Notably, *C. pseudotuberculosis* was not recovered from any animal-associated or environmental sample under the culture conditions used, as the profiling was not designed as a targeted assay for this fastidious pathogen. Consequently, farm-level associations between environmental *C. pseudotuberculosis* burden and vaccine-related outcomes could not be tested, and the profiling component is presented as a purely descriptive characterization of the culturable microbial background rather than as a hypothesis-testing analysis. This distinction is important because culture-based profiling is influenced by sample type, culture medium, incubation condition, representative colony selection, and database coverage [28,29,30].

Targeted PCR provided pathogen-specific information that complemented the culture-based profiling. *Pasteurella multocida* and *Mannheimia haemolytica* were detected in nasal swabs, while bovine rotavirus and bovine coronavirus were detected in rectal swabs. These organisms are not etiological agents of CLA, but their detection indicates that the enrolled farms differed in respiratory and enteric pathogen pressure. *P. multocida* and *M. haemolytica* are commonly investigated in respiratory disease of goats, while bovine coronavirus and rotavirus have been detected in small-ruminant enteric disease investigations [19,26,31,32,33]. Therefore, Table 2 should be interpreted as a management-relevant pathogen summary rather than as an explanatory table for CLA vaccine efficacy. This table complements the broader culture/MALDI-TOF MS isolate profile by identifying selected organisms that may influence farm-level health management and interpretation of field observations.

For field application in Korean goat farms, CLA vaccination should be used as part of an integrated control strategy. The Korean native black goat industry has expanded, but recent reviews have emphasized persistent gaps in goat-specific vaccines, therapeutics, veterinary support, surveillance, and structured herd-health management [13]. Vaccination alone is unlikely to eliminate CLA from contaminated herds because *C. pseudotuberculosis* can persist in abscess material and on farm-associated fomites [5,6]. Environmental survival studies have shown that the organism can remain viable in purulent exudate and common barnyard materials, supporting the need for sanitation and abscess-management measures in addition to vaccination [5,6]. Field control programs also require detection of infected or exposed animals, appropriate handling of abscess-positive goats, hygiene management during lesion rupture, and reduction in environmental contamination [1,2,3,20,40]. The present field data are consistent with this integrated-control concept: the vaccine induced measurable antibody responses at the vaccination-trial farms, while bacteriological profiling across the surveyed farms revealed heterogeneous microbial backgrounds. These two observations are presented as complementary field context rather than as a causal link between farm-level microbial burden and vaccine outcome.

Several limitations should be considered when interpreting these findings. First, the number of confirmed external abscess cases was small, limiting statistical power for the clinical endpoint. Second, the trial was conducted under natural exposure conditions without experimental challenge, so exposure dose and timing were not controlled. Third, internal abscesses were not systematically assessed by necropsy or imaging, and clinical outcome assessment was limited to externally observed lesions; because internal disease may occur without external signs, the true CLA burden may have been underestimated, and clinical protection cannot be inferred from external observations alone. Fourth, although the in-house ELISA was optimized and showed strong agreement with the commercial assay (Supplementary Figure S1), full analytical validation (inter- and intra-assay coefficients of variation, analytical specificity, and diagnostic sensitivity and specificity) was not performed; the assay was therefore used for longitudinal monitoring only and should not be used for diagnostic purposes without such validation. Fifth, culture/MALDI-TOF MS profiling captured culturable organisms only and should not be interpreted as a full microbial community analysis. These limitations should be considered in future studies designed to evaluate clinical protection, long-term herd outcomes, and environmental contamination risk. In addition, clinical observations were recorded by farm owners under veterinary supervision rather than by independent, blinded investigators. Although veterinary supervision, standardized scoring criteria, and prior joint training were used to reduce inter-observer variability, formal inter-observer reliability was not quantified, and some degree of observer-related variability cannot be excluded in field-based evaluations. In addition, because each farm received a single vaccine lot, vaccine lot and farm were confounded, and lot-specific effects could not be separated from farm-level differences. In addition, because the study was conducted on commercial farms, animals were not blocked or balanced by breed or sex across groups, and breed and sex composition varied by farm; group assignment was based on baseline serostatus rather than on randomization with stratification. These field constraints should be considered when interpreting between-group comparisons.

In summary, this field study extended the previous experimental evaluation of a Korean inactivated CLA vaccine into commercial goat farm conditions [11]. The vaccine induced CLA-specific antibody responses in both commercial CLA ELISA and in-house whole-bacterial IgG ELISA assays and was not associated with a persistent reduction in growth performance. External abscess occurrence was limited to baseline seropositive animals. The difference between vaccinated and non-vaccinated seropositive goats was not statistically significant, and the clinical endpoint was underpowered; it is therefore reported as exploratory rather than as evidence of clinical protection. Culture/MALDI-TOF MS and targeted PCR further showed that farm-level microbial backgrounds differed across the enrolled farms. These findings support considering CLA vaccination as one component of a broader control program that includes serological monitoring, clinical surveillance, abscess management, and environmental hygiene.

## 5. Conclusions

This field study extended the evaluation of a Korean inactivated CLA vaccine to commercial goat farm conditions. Vaccination induced CLA-specific antibody responses in both the commercial CLA ELISA and the in-house whole-bacterial IgG ELISA, and was not associated with a persistent reduction in growth performance. External abscess formation was limited to baseline seropositive animals; however, the difference between vaccinated and non-vaccinated seropositive goats was not statistically significant, and the clinical endpoint was underpowered, so no conclusion regarding clinical protection can be drawn. Culture/MALDI-TOF MS and targeted PCR further showed that farm-level microbial backgrounds differed across the enrolled farms, reinforcing the need to interpret CLA vaccine performance within the context of natural exposure conditions rather than in isolation. These findings support the evaluation of CLA vaccination as one component of an integrated control strategy that includes serological monitoring, clinical surveillance, abscess management, and environmental hygiene. Building on these results, future studies should incorporate larger, adequately powered sample sizes to assess clinical protection, systematic assessment of internal lesions by necropsy or imaging, longer post-vaccination follow-up, and targeted environmental sampling for *C. pseudotuberculosis* specifically, to better link vaccine-induced responses to disease outcomes under field conditions.

## 6. Patents

A Korean patent has been registered for the vaccine strain and composition described in this study: “Novel *Corynebacterium pseudotuberculosis* strain isolated from a caseous lymphadenitis-infected goat and vaccine composition for preventing caseous lymphadenitis comprising the same” (Korean Patent No. KR10-2963232).

## Figures and Tables

**Table 2:** Detection of selected respiratory, enteric, and environmental organisms by targeted PCR and culture/MALDI-TOF MS.

Target Organism/ Pathogen	Main Sample Type	Method	No. Positive /No. Tested	Farm-Level Detection	Interpretation
*Pasteurella multocida*	Nasal swab	PCR, Culture MALDI-TOF MS	4/22 by PCR	A, D, E	Detected in selected farms
*Mannheimia haemolytica*	Nasal swab	PCR	7/22	A, B, D, F	Respiratory pathogen detected by PCR
*Streptococcus pluranimalium*	Nasal swab	Culture MALDI-TOF MS	NA	A, D	Opportunistic respiratory-associated bacterium
*Staphylococcus aureus*	Nasal swab	Culture MALDI-TOF MS	NA	C, D, F	Potential asymptomatic carriage
*Clostridium septicum*	Barn-floor soil	Culture MALDI-TOF MS	NA	F	Environmental pathogen-associated bacterium
Bovine rotavirus	Rectal Swab	PCR	4/19	A, B, C	Sporadic detection
Bovine coronavirus	Rectal Swab	PCR	9/19	A, B, C, F	Farm-dependent detection

NA, not applicable because culture/MALDI-TOF MS results were summarized by detected isolate distribution rather than by target-specific PCR denominator. PCR positivity was determined by target-specific amplification and agarose gel electrophoresis.

## Data Availability

Data supporting the findings of this study are available from the corresponding author upon reasonable request. Farm-identifying information and individual animal records are not publicly available to protect the confidentiality of participating farms.

## Supplementary Materials

The following supporting information can be downloaded at: https://www.mdpi.com/article/10.3390/vaccines14080695/s1, Figure S1: Optimization of antigen coating concentration, blocking condition, serum dilution, and preliminary comparative assessment between the in-house CLA-specific IgG ELISA and the commercial CLA ELISA kit.

## Abbreviations

The following abbreviations are used in this manuscript:

AWWG	Average weekly weight gain
BHI	Brain heart infusion
CFU	Colony-forming unit
CLA	Caseous lymphadenitis
DNA	Deoxyribonucleic acid
DPV	Days post-vaccination
ELISA	Enzyme-linked immunosorbent assay
HE	hemagglutinin esterase
HRP	Horseradish peroxidase
IACUC	Institutional animal care and use committee
IgG	Immunoglobulin G
KNBG	Korean native black goat
MALDI-TOF MS	Matrix-assisted laser desorption/ionization of flight mass spectrometry
OD_450_	Optical density at 450 nm
PBS	Phosphate-buffered saline
PBST	Phosphate-buffered saline containing Tween 20
PCR	Polymerase chain reaction
RNA	Ribonucleic acid
SD	standard deviation
SEM	Standard error of the mean
SN	Seronegative
SN-Vac	Seronegative vaccinated
SP	Seropositive
SP-Vac	Seropositive vaccinated
SP-NonVac	Seropositive non-vaccinated
S/P	sample-to-positive ratio
TMB	3,3′,5,5′-tetramethylbenzidine
WPV	Weeks post-vaccination
