# Supplementary material for: Integrated Field Evaluation of a Caseous Lymphadenitis Vaccine and Environmental Bacteriological Profiling in Commercial Goat Farms in South Korea
Source: Vaccines (Basel). 2026 Aug 12;14(8):695. doi: 10.3390/vaccines14080695 (PMC13517150; doi:10.3390/vaccines14080695)
Supplement: Supplementary file 1 [file vaccines-14-00695-s001.zip › vaccines-4441340-supplementary.pdf]

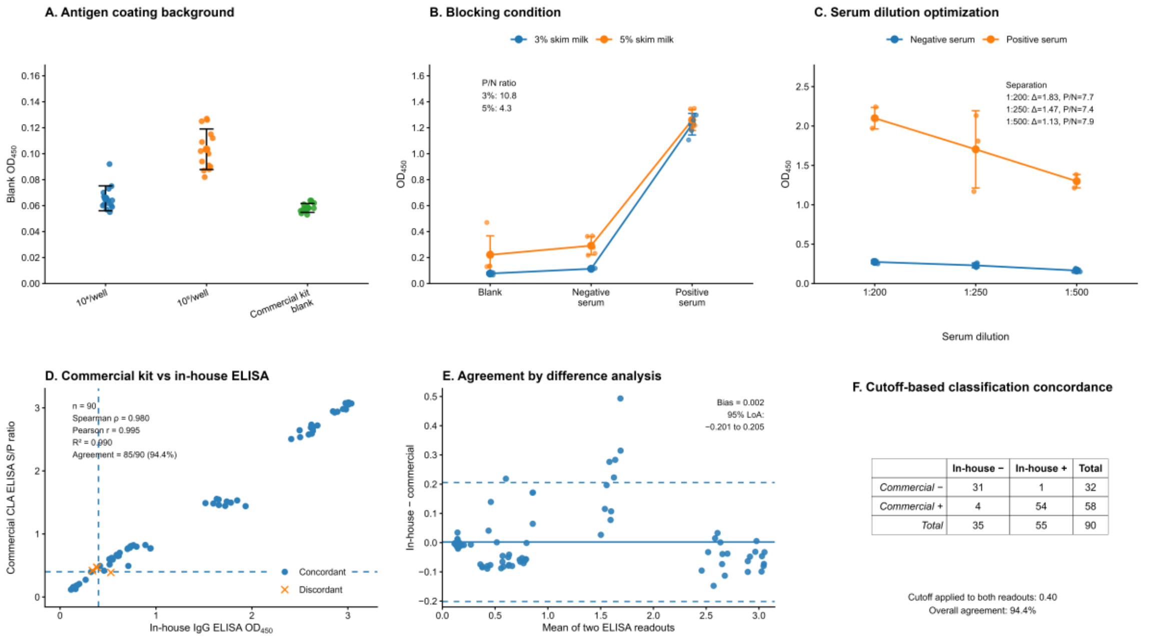

Supplementary Figure S1. Optimization and preliminary comparative assessment of the in-house CLA-specific IgG ELISA. (A) Antigen coating background was evaluated by comparing blank OD_{450} values obtained using 10^4 CFU-equivalent/well and 10^5 CFU-equivalent/well inactivated whole-bacterial antigen coating conditions and the commercial CLA ELISA kit blank. Points indicate individual replicate values, and horizontal markers indicate mean ± SD. (B) Blocking conditions were compared using 3% and 5% skim milk in PBS. Blank, negative-serum, and positive-serum OD_{450} values are shown. The positive-to-negative (P/N) ratio was 10.8 for 3% skim milk and 4.3 for 5% skim milk. (C) Serum dilution was optimized by comparing negative and positive goat serum responses at 1:200, 1:250, and 1:500 dilutions. The 1:500 dilution reduced negative-serum background while maintaining positive-negative separation. (D) Paired comparison between the in-house whole-bacterial IgG ELISA and the commercial CLA ELISA kit using 90 serum samples. Dashed lines indicate the cutoff value of 0.40 applied to both readouts. The in-house ELISA showed strong agreement with the commercial kit, with Spearman's rho = 0.980, Pearson r = 0.995, $R^2 = 0.990$, and 85/90 classification agreement (94.4%). (E) Bland–Altman-style difference analysis showing the difference between in-house and commercial ELISA readouts plotted against the mean of the two readouts. The mean difference was 0.002, with 95% limits of agreement from −0.201 to 0.205. (F) Cutoff-based classification concordance table for the paired comparison shown in panel D. CLA, caseous lymphadenitis; ELISA, enzyme-linked immunosorbent assay; IgG, immunoglobulin G; OD_{450}, optical density at 450 nm; PBS, phosphate-buffered saline; SD, standard deviation; S/P, sample-to-positive ratio.
